# Differential Diagnosis of Merc. Viv. and Podophyl.

**Published:** 1881-02

**Authors:** C. Carleton Smith

**Affiliations:** Phila.


### DIFFERENTIAL DIAGNOSIS OF MERC. VIVUS AND PODOPHYLLUM.

C. CARLETON SMITH, M.D., PHILA.

| *Murcurios Viv.* | *Popophyllum.* |
|---|---|
| **MENTAL.** | |
| FEARS he will surely lose his reason, and that he will become insane. | Low spirited, fears he will die. Becomes hypochondrical if his illness lasts long. |
| **HEAD.** | |
| Frontal headache as if head would split open, with much fullness and heat, in the evening or later in the night. Headache as soon as he lies down at night; has to sit in chair, with sore mouth and aching in bones of extremities. | Temples throb, hot head and eyes ache, especially in the morning. Rolling of head from side to side. Child grinds its teeth and whines, especially at night. Slow dentition. |
| **EYES.** | |
| Pustules appear on the cornea, lids become crusty; agg. eve. and night. | Scrofulous opthalmia always worse in the morning. |
| **MOUTH.** | |
| Very foetid breath. Tongue quite yellow *all over*, (*merc. iod.* only yellow at *root*) very moist and flabby. Saliva foetid and profuse. | Breath foul, with white, dry tongue. Saliva copious, but not foetid. |
| **THROAT.** | |
| Throat very sore; left side generally worse; agg. from swallowing saliva or liquids; agg. from exposure to evening air. | Throat sore; worse in right side, and in the morning, and swallowing liquids. |

STOMACH.

Nausea, vomiting bile, having *bitter* or a *sweet* taste. Fullness in stomach at times but flatus not quite so marked as in Pod.

Nausea with gagging and retching; vomits blood; retching is very painful.

ABDOMEN.

Liver so very sensitive to pressure he can not lie on right side, nor bear the least touch of finger. Costiveness with ineffectual straining. Colic very similar to worm colic, as we find it in children with hard, tense abdomen. Stools are small and crumble; sometimes *black*, sometimes gray approaching white; again they are watery corrosive, slimy, bloody, with much tenesmus; never guishing either sour or entirely odorless. Agg. eve. and night, until 3 A.M. (Time similar to *rhus. tox*). After stool great tenesmus with cutting pain. Sour sweat, trembling of body, and burning in anus. Rectum becomes prolapsed, and so inflamed in some cases as to become *black* from the terrible straining which the patient can not avoid.

Flatulency confined to *right* side of abdomen; palpitation with morning drowsiness. Liver feels hot and becomes sore with twisting pains; relief from rubbing over region of liver. Constipation, dry stools, and quite hard. Stools chalky and very offensive with gagging, profuse, painless, gushing stool, greenish, or yellow, or mixed with blood smelling like carrion; worse in the morning. After stool the patient is greatly exhausted, and pains continue of a cutting nature, weakness even after a natural stool. Prolapsus recti with the diarrhœa and from the least exertion. (The foulness of Pod. is almost indescribable, and not to be compared with any other drug unless it may be *psorinum*.

URINE.

Scantiness of urine, with much urging (similar to *nux*).

Urine quite scanty, but frequent, especially at night and during pregnancy.

GENITALS.

Shooting pains from both ovaries toward the hips. Vagina becomes prolapsed and also uterus. Soreness of the genitals internally and externally. During pregnancy, stomach becomes very sensitive to touch or extra pressure of clothing accompanied with sore mouth, tender gums and teeth.

Dragging pains in ovarian regions. Prolapsus uteri with severe backache over region of sacral bone, especially after confinement and from standing over the washtub.

During gestation can lie only on the abdomen especially the first three or four months; passes water often, followed by prolapsus of womb.

CHEST.

Cough with sensation of burning and rawness of the bronchia, but no phlegm is expectorated; cough comes in convulsive paroxysms preventing conversation on the part of the sufferer. Whooping-cough comes in two distinct paroxysms and then a rest.

On awaking, great agitation of the heart causing the patient to feel that death was imminent.

Cough loose during dentition. Palpitation from any exertion, accompanied with troublesome flatulency; heart feels as if moving upward into the throat.

FEVER.

Pulse generally full and strong; chill mostly in the evening, sometimes 5 A.M., on getting out of bed. With the heat there is constriction of chest. Sweat usually cold, and gives no relief to existing symptoms. Patient gets chilly after

Pulse slow; sometimes can hardly be felt; almost collapsed. Chill 7 A.M.

Heat accompanied with delerium and talkativeness. Soon forgets what has passed.

Sweet on the legs which is warm while the feet are cold:

MERC.

a stool. Agg. from draft of cold air coming in contact with body through seat of out door water closet.

PODO.

cold sweat of head; sweats while she sleeps. (*China.*)

AGGRAVATION.

MERC.

Worse from making any sort of motion; worse eve. and night and after getting warm in bed (rheumatism). Agg. from *sweet things*—candy.

PODO.

Worse from traveling over uneven ground, and from making mis-steps (*bry.*) Worse usually mornings. Worse from sour fruit, especially when combined with milk.

